# Prematurity is associated with altered central thyroid hormone sensitivity

**DOI:** 10.3389/fendo.2026.1896019

**Published:** 2026-08-19

**Authors:** Gülçin Arslan, Hakan Birinci, Şükrü Onur Halaç, Eren Er, Aslıhan Abbasoğlu, Bumin Nuri Dündar

**Affiliations:** 1 Department of Pediatric Endocrinology, İzmir City Hospital, İzmir, Türkiye; 2 Department of Neonatology, İzmir City Hospital, İzmir, Türkiye; 3 Department of Neonatology, İzmir Katip Çelebi University Faculty of Medicine, İzmir, Türkiye; 4 Department of Pediatric Endocrinology, İzmir Katip Çelebi University Faculty of Medicine, İzmir, Türkiye

**Keywords:** neonate, prematurity, TFQI, thyroid function tests, TSHI, TT4RI

## Abstract

**Objectives:**

Thyroid Feedback Quantile-based Index (TFQI), the Thyrotropin–Thyroxine Resistance Index (TT4RI), and the Thyrotropin Index (TSHI) have been developed to assess central sensitivity to thyroid hormones by integrating circulating thyroid hormone levels with the pituitary thyrotropin response. This study aimed to investigate central and peripheral thyroid hormone sensitivity in preterm neonates using established thyroid hormone sensitivity indices.

**Methods:**

This study included 70 neonates monitored in our hospital’s neonatal intensive care unit (NICU), categorized into two groups: those born at <32 weeks of gestation (n=17) and those born at ≥32 weeks (n=53). Clinical characteristics and thyroid function test results were recorded. Peripheral thyroid hormone sensitivity was assessed using the Free T3 (FT3)/Free T4 (FT4) ratio, while central thyroid hormone sensitivity was evaluated using TT4RI, TSHI, and TFQI, with lower FT3/FT4 and higher TT4RI, TSHI, and TFQI values indicating reduced thyroid hormone sensitivity.

**Results:**

The mean postnatal age of neonates born at <32 weeks (n=17; 9 females, 52.9%) was 92.50 ± 21.34 days, compared with 58.09 ± 20.21 days for those born at ≥32 weeks (n=53; 26 females, 49.0%). TSHI, TT4RI, and TFQI, were significantly lower in neonates born at <32 weeks compared with those born at ≥32 weeks.

**Conclusions:**

Lower thyroid hormone sensitivity indices in early preterm neonates suggest altered central thyroid hormone regulation despite normal conventional thyroid function tests. These findings underscore the clinical importance of incorporating sensitivity indices into the evaluation of preterm neonates and suggest that further monitoring of their metabolic and cardiovascular status is warranted.

## Introduction

Thyroid hormones play a crucial role in fetal growth, brain maturation, and metabolic regulation, particularly during the perinatal period (1, 2). During intrauterine life, a significant portion of fetal thyroxine (T4) is derived from maternal transfer, especially in early gestation before the full maturation of the fetal hypothalamic–pituitary–thyroid (HPT) axis (1). In term neonates, delivery is followed by a rapid surge in thyroid-stimulating hormone (TSH), which peaks within the first hours of life and subsequently stimulates a marked increase in circulating T4 and triiodothyronine (T3) levels (2). This physiological transition is essential for postnatal adaptation.

In contrast, preterm infants exhibit a distinctly different pattern of thyroid function due to the immaturity of the HPT axis (1, 3). The postnatal TSH surge is often blunted, and the subsequent rise in T4 and T3 levels is attenuated compared to term infants (3). As a result, circulating thyroid hormone levels are generally lower and show a strong positive correlation with gestational age and birth weight (1). A characteristic feature of this population is transient hypothyroxinemia of prematurity (THOP), defined by low serum T4 concentrations alongside normal or low TSH levels (3, 4). This condition is multifactorial in origin, reflecting the combined effects of HPT axis immaturity, reduced thyroidal hormone synthesis, decreased thyroxine-binding globulin levels, increased peripheral metabolism, and the impact of non-thyroidal illness (3, 4). Several external factors further influence thyroid function in this vulnerable group, including iodine imbalance, medications such as dopamine or glucocorticoids, and the severity of concurrent systemic illness (3, 5). Given these unique physiological and pathological characteristics, the interpretation of thyroid function tests in preterm neonates requires careful consideration of gestational age–specific reference ranges and longitudinal monitoring (1, 4). Understanding these differences is essential for optimizing screening strategies and ensuring timely diagnosis and management of thyroid dysfunction in preterm infants.

Previous studies have suggested that subclinical thyroid hormone resistance may exist within the general population, and a novel metric, termed the Thyroid Feedback Quantile-based Index (TFQI), has been proposed to assess deviations of the pituitary response from the population-based average thyroid hormone-mediated suppression. Notably, TFQI has demonstrated superior clinical applicability and diagnostic performance compared to earlier indices of central resistance, including the Thyrotroph Thyroxine Resistance Index (TT4RI) and the Thyroid-Stimulating Hormone Index (TSHI) (6). Peripheral thyroid hormone sensitivity can be approximated by the FT3/FT4 ratio, whereas central sensitivity can be assessed using indices such as the TFQI, the TT4RI, and the TSHI (7). In recent years, studies examining the association between thyroid hormone sensitivity and various diseases have gained increasing attention (8, 9); however, data in pediatric populations remain scarce (10, 11). The interpretation of thyroid function tests (TFTs) is challenging, as circulating hormone levels may fall within reference ranges despite altered tissue-level thyroid hormone action in the neonatal period, particularly in preterm infants. Therefore, apparently ‘normal’ TFT results may not necessarily indicate normal thyroid hormone sensitivity in this vulnerable population. To the best of our knowledge, no previous studies have specifically evaluated thyroid hormone sensitivity in newborns, especially in preterm infants. Based on this premise, the present study aimed to assess thyroid hormone sensitivity in preterm and term neonates using established central and peripheral sensitivity indices.

## Materials and methods

### Study population

This retrospective cross-sectional study with a case–control design was conducted at the pediatric endocrinology and neonatology clinics of Izmir City Hospital between 31 December 2025 and 1 April 2026. The study was approved by the local Ethics Committee of Izmir Katip Çelebi University (ethical committee date: 06/11/2025; ethical committee decision no: 0662), and informed consent was obtained from the parents of all subjects.

This study included 70 neonates monitored in the neonatal intensive care unit of our hospital, comprising 17 infants born at <32 weeks of gestation and 53 infants born at ≥32 weeks of gestation. TFTs are routinely performed as part of the standard follow-up protocol for all hospitalized neonates at the relevant postnatal age in our neonatal intensive care unit. Infants born before 32 weeks represent the group at highest risk for hypothalamic-pituitary-thyroid axis immaturity and transient hypothyroxinemia of prematurity (12). Therefore, this threshold is widely used in neonatal endocrinology and was selected *a priori*. All consecutive eligible neonates with available TFTs meeting the predefined inclusion criteria during the study period were included. Exclusion criteria consisted of neonates with:

- Systemic disorders or acute serious infections.
- A history of thyroid hormone replacement therapy.
- Use of medications known to affect thyroid function.

Clinical and anthropometric characteristics, thyroid function test results, and calculated indices of thyroid hormone sensitivity were recorded.

The sample size was determined by the number of neonates who met the inclusion criteria and were admitted to the neonatal intensive care unit during the study period. A *post hoc* power analysis, based on the observed TFQI difference between groups, indicated that the study had sufficient statistical power (>99%) to detect the observed effect size (Cohen’s d ≈ 1.57) at a two-sided alpha level of 0.05.

### Study variables

Demographic characteristics (age and sex), anthropometric measurements, and thyroid function test results were extracted from the medical records of the patients. Body proportionality was assessed using the triponderal index (TPI), calculated as weight in kilograms divided by height in meters cubed (kg/m³). In the neonatal period, normal reference ranges for thyroid function tests are determined based on both gestational age and postnatal age, reflecting the dynamic maturation of the hypothalamic–pituitary–thyroid axis (13).

### Calculation of thyroid hormone sensitivity

Peripheral thyroid hormone sensitivity was assessed using the free triiodothyronine to free thyroxine ratio (FT3 [pg/mL]/FT4 [pmol/L]). A lower FT3/FT4 ratio was considered indicative of reduced peripheral thyroid hormone sensitivity (14). Central thyroid hormone sensitivity was evaluated using three established indices: the TT4RI, the TSHI, and the TFQI. TT4RI was calculated as the product of FT4 (pmol/L) and TSH (mIU/L) (7). TSHI was calculated using the formula ln[TSH (mIU/L)] + [0.1345 × FT4 (pmol/L)] (7). TFQI was calculated as the difference between the cumulative distribution function of FT4 and one minus the cumulative distribution function of TSH [TFQI = cdf(FT4) − (1 − cdf(TSH))] (8). Higher TT4RI and TSHI values, as well as positive TFQI values, were interpreted as indicators of reduced central thyroid hormone sensitivity, reflecting impaired hypothalamic–pituitary feedback to circulating thyroid hormones (7, 8).

### Statistical analysis

Statistical analysis was performed using SPSS version 26.0. Descriptive data are presented as frequencies and percentages (%) for categorical variables and as mean ± standard deviation (SD) or median (interquartile range [IQR]) for continuous variables. The normality of each continuous variable was assessed individually using the Shapiro–Wilk test. Variables with a normal distribution are presented as mean ± standard deviation (SD), whereas non-normally distributed variables are presented as median (interquartile range). Appropriate parametric or non-parametric tests were applied accordingly. Group comparisons were conducted using the chi-square or Fisher’s exact test for categorical variables, and the Student’s t-test, Mann–Whitney U test, or Kruskal–Wallis test for continuous variables, as appropriate. Correlations between continuous variables were analyzed using Spearman’s ρ coefficient. A p-value < 0.05 was considered statistically significant. To account for multiple comparisons among the three central thyroid hormone sensitivity indices, a Bonferroni-corrected significance threshold of p < 0.0167 was applied (0.05/3).

## Results

The final study population consisted of 70 neonates after applying the exclusion criteria. The mean postnatal age of neonates born at <32 weeks (n=17; 9 females, 52.9%) was 92.50 ± 21.34 days, compared with 58.09 ± 20.21 days in those born at ≥32 weeks (n=53; 26 females, 49.0%). Body weight, height, and TPI values of the participants are presented in **Table 1**. FT3 and FT4 levels, as well as TSHI, TT4RI, and TFQI, were significantly lower in neonates born at <32 weeks compared with those born at ≥32 weeks, whereas the FT3/FT4 ratio, reflecting peripheral thyroid hormone sensitivity, was similar between the two groups (**Table 2**). These findings suggest increased central thyroid hormone sensitivity in early preterm neonates, while peripheral hormone sensitivity appears to be preserved. The differences in TT4RI, TSHI, and TFQI remained statistically significant after Bonferroni correction, using a corrected significance threshold of p < 0.0167.

**Table 1:** Comparison of demographic and anthropometric characteristics of neonates according to gestational age groups.

Variables	<32 weeks of gestation (n = 17)	≥32 weeks of gestation (n = 53)	*P* value
Age (days), mean ± SD	92.50 ± 21.34	58.09 ± 20.21	**<0.001**
Female sex, n (%)	9 (52.9)	26 (49.0)	1.000
Body weight (g), mean ± SD	1469.12 ± 329.83	2691.32 ± 796.46	**<0.001**
Length (cm), mean ± SD	40.11 ± 3.49	47.19 ± 4.78	**<0.001**
Triponderal index, mean ± SD	2.27 ± 0.37	2.54 ± 0.61	0.090

SD, standard deviation. Bold values indicate statistical significance (p < 0.05).

**Table 2:** Comparison of thyroid function tests and thyroid hormone sensitivity indices according to gestational age groups.

Variables	<32 weeks of gestation (n = 17)	≥32 weeks of gestation (n = 53)	*P* value
Thyroid function tests			
TSH, mean ± SD	2.65 ± 1.59	4.87 ± 3.70	**0.001**
fT4, mean ± SD	15.09 ± 3.40	21.20 ± 4.47	**<0.001**
fT3, mean ± SD	2.23 ± 0.63	3.61 ± 1.02	**<0.001**
fT3/fT4 ratio, mean ± SD	0.15 ± 0.04	0.17 ± 0.04	0.119
Thyroid hormone sensitivity indices			
TT4RI, mean ± SD	40.07 ± 28.41	101.48 ± 74.99	**<0.001**
TSHI, mean ± SD	4.68 ± 1.65	7.73 ± 1.67	**<0.001**
TFQI, mean ± SD	−0.41 ± 0.32	0.14 ± 0.38	**<0.001**

SD, standard deviation; TSH, thyroid-stimulating hormone; fT4, free thyroxine; fT3, free triiodothyronine; TT4RI, thyrotropin–thyroxine resistance index; TSHI, thyrotropin index; TFQI, thyroid feedback quantile-based index. Bold values indicate statistical significance (p < 0.05).

To account for variation in the timing of thyroid function testing, an ANCOVA was performed with postmenstrual age (PMA) at blood sampling included as a covariate. PMA was not significantly associated with TFQI (p = 0.941), Serum TT4RI (p = 0.453), or Serum TSHI (p = 0.665). After adjustment for PMA, significant differences between gestational age groups remained for TFQI (F = 13.563, p < 0.001, ηp² = 0.170), Serum TT4RI (F = 7.555, p = 0.008, ηp² = 0.103), and Serum TSHI (F = 6.358, p = 0.014, ηp² = 0.088). Compared with neonates born at <32 weeks of gestation, those born at ≥32 weeks had significantly higher adjusted mean TFQI, Serum TT4RI, and Serum TSHI values (**Table 3**).

**Table 3:** Comparison of adjusted thyroid hormone sensitivity indices between very preterm and moderate-to-late preterm neonates using ANCOVA.

Dependent variable	Group 0 (≥32 weeks) adjusted mean ± SE	Group 1 (<32 weeks) adjusted mean ± SE	F (group)	P (group)	Partial Eta Squared (ηp²)	Covariate p (PMA)
TFQI	0.147 ± 0.058	−0.424 ± 0.128	13.563	<0.001	0.170	0.941
Serum TT4RI	104.996 ± 10.394	28.426 ± 22.895	7.555	0.008	0.103	0.453
Serum TSHI	7.832 ± 0.515	4.351 ± 1.135	6.358	0.014	0.088	0.665

*Values are presented as adjusted means ± standard error (SE). Analyses were performed using ANCOVA with postmenstrual age (PMA) as the covariate. Adjusted means are estimated at the overall mean PMA of 36.28 weeks. SE, standard error; PMA, postmenstrual age; TFQI, Thyroid Feedback Quantile-based Index; TT4RI, Thyrotroph T4 Resistance Index; TSHI, Thyroid-Stimulating Hormone Index.

## Discussion

To our knowledge, this is the first study to evaluate the relationship between thyroid hormone sensitivity and gestational age. We demonstrated that central thyroid hormone sensitivity—as reflected by TSHI, TT4RI, and TFQI—significantly differs according to gestational age in neonates. Conversely, the FT3/FT4 ratio, an indicator of peripheral thyroid hormone sensitivity, did not differ significantly between the two groups. This suggests that while central regulation is age-dependent, peripheral thyroid hormone sensitivity remains relatively preserved across gestational age groups. In this study, preterm infants born at <32 weeks of gestation exhibited significantly lower body weight and length compared to those born at ≥32 weeks, consistent with the established impact of gestational age on somatic growth (15). These findings reflect the degree of physiological immaturity and growth restriction commonly observed in very premature neonates (16). Despite these differences in anthropometric measurements, TPI, a marker of body proportionality, was comparable between the groups. This indicates that while preterm infants had lower absolute weight and length, their overall body proportionality was preserved, suggesting that growth impairment in this cohort is proportionate rather than disproportionate during the early neonatal period (17). Furthermore, the preterm group had a significantly higher postnatal age at the time of evaluation. This discrepancy likely reflects clinical follow-up practices in neonatal intensive care units (NICUs), where more premature infants require prolonged hospitalization and monitoring. Given that thyroid function is dynamic and influenced by postnatal age, this difference must be considered when interpreting thyroid-related findings (12, 18). No significant difference was observed in sex distribution, suggesting that the biochemical variations observed are unlikely to be confounded by sex-related factors. Our results showed that infants born at <32 weeks had significantly lower TSH, FT3 and FT4 levels compared to those born at ≥32 weeks, which aligns with the known immaturity of the HPT axis in premature neonates (19). Reduced TSH secretion, alongside lower circulating thyroid hormone levels, likely reflects an underdeveloped pituitary response and limited thyroidal capacity at earlier gestational ages. The lower FT3 and FT4 levels in the <32-week group are consistent with THOP, a condition attributed to both decreased hormone production and reduced peripheral conversion (19). Despite these absolute hormonal differences, the comparable FT3/FT4 ratio suggests that the peripheral conversion of FT3 and FT4 remains functional. Therefore, the primary alteration in preterm infants appears to be localized to the central regulation of the HPT axis rather than peripheral metabolism. The significant differences in TT4RI, TSHI, and TFQI further support this. Preterm infants (<32 weeks) exhibited significantly lower values across these indices. The TFQI, a recently developed and robust marker, identifies subtle alterations in thyroid hormone feedback regulation even in euthyroid individuals (8). Our findings indicate increased pituitary responsiveness to circulating thyroid hormones in more premature neonates. Lower TFQI values in the preterm group may indicate an immature or downregulated central feedback mechanism, consistent with the developmental immaturity of the HPT axis (16). These results suggest a dissociation between central and peripheral thyroid hormone sensitivity. While peripheral responsiveness appears preserved, central regulatory mechanisms are significantly altered by prematurity. This distinction is clinically vital, as conventional TFTs alone may not fully capture the nuances of thyroid hormone action in this population.

Notably, the associations remained significant after adjustment for postmenstrual age, suggesting that the lower thyroid hormone sensitivity indices in neonates born at <32 weeks were not merely attributable to differences in blood sampling time. Instead, these findings support the concept that very preterm birth is associated with immature hypothalamic–pituitary–thyroid axis regulation, consistent with the pathophysiology of THOP.

This study has several limitations. First, the retrospective and single-center design may have introduced selection bias and limited our ability to control for all potential confounding factors. Second, the sample size was determined by available records. Although a *post hoc* power analysis demonstrated excellent statistical power for the primary outcome (TFQI), this finding should be interpreted cautiously because *post hoc* power is dependent on the observed effect size and does not overcome the limitations associated with a relatively small sample size. Furthermore, our study was not prospectively powered to detect clinically meaningful differences in secondary outcomes or to support subgroup analyses. Therefore, the absence of a statistically significant difference in the FT3/FT4 ratio should not be interpreted as evidence of preserved peripheral thyroid hormone sensitivity, as the study may have been underpowered to detect small-to-moderate differences. The relatively small number of infants born before 32 weeks reflects the lower frequency of extremely preterm births in our center. In addition, we did not evaluate neurodevelopmental, metabolic or growth outcomes; therefore this study does not allow conclusions to be drawn regarding the prognostic value of the thyroid hormone sensitivity indices. Prospective studies including clinically relevant outcomes will be necessary to determine whether these indices are associated with short- and long-term clinical outcomes.

## Conclusions

In conclusion, our findings demonstrate that thyroid hormone sensitivity indices—particularly the TFQI—capture clinically relevant alterations in central thyroid hormone regulation in neonates. These changes are more pronounced in preterm infants, highlighting that conventional TFTs may be insufficient to fully reflect thyroid signaling during the neonatal period. Our study provides novel evidence that thyroid hormone sensitivity is modified by gestational immaturity, underscoring the importance of incorporating sensitivity-based indices into neonatal endocrine evaluations. These findings provide a strong rationale for future longitudinal studies to elucidate the role of thyroid sensitivity in neonatal adaptation and long-term developmental outcomes.

## Data Availability

The original contributions presented in the study are included in the article/supplementary material. Further inquiries can be directed to the corresponding author.
